# Decreased HIV diversity after allogeneic stem cell transplantation of an HIV-1 infected patient: a case report

**DOI:** 10.1186/1743-422X-7-55

**Published:** 2010-03-08

**Authors:** Christel Kamp, Timo Wolf, Ignacio G Bravo, Benjamin Kraus, Birgit Krause, Britta Neumann, Gudrun Winskowsky, Alexander Thielen, Albrecht Werner, Barbara S Schnierle

**Affiliations:** 1 Paul-Ehrlich-Institut, Paul-Ehrlich-Strasse 51-59, 63225 Langen, Germany; 2 Johann-Wolfgang-Goethe University, Medical School, Theodor-Stern-Kai 7, Frankfurt am Main, Germany; 3 European Commission, Directorate Health and Consumer Protection, Health Threat Unit C3, Luxembourg; 4 Centro Superior de Investigación en Salud Pública, CSISP, Valencia, Spain; 5 Max-Planck-Institut für Informatik, Campus E14, 66123 Saarbrücken, Germany

## Abstract

The human immunodeficiency virus type 1 (HIV-1) coreceptor use and viral evolution were analyzed in blood samples from an HIV-1 infected patient undergoing allogeneic stem cell transplantation (SCT). Coreceptor use was predicted *in silico *from sequence data obtained from the third variable loop region of the viral envelope gene with two software tools. Viral diversity and evolution was evaluated on the same samples by Bayesian inference and maximum likelihood methods. In addition, phenotypic analysis was done by comparison of viral growth in peripheral blood mononuclear cells and in a CCR5 (R5)-deficient T-cell line which was controlled by a reporter assay confirming viral tropism. *In silico *coreceptor predictions did not match experimental determinations that showed a consistent R5 tropism. Anti-HIV directed antibodies could be detected before and after the SCT. These preexisting antibodies did not prevent viral rebound after the interruption of antiretroviral therapy during the SCT. Eventually, transplantation and readministration of anti-retroviral drugs lead to sustained increase in CD4 counts and decreased viral load to undetectable levels. Unexpectedly, viral diversity decreased after successful SCT. Our data evidence that only R5-tropic virus was found in the patient before and after transplantation. Therefore, blocking CCR5 receptor during stem cell transplantation might have had beneficial effects and this might apply to more patients undergoing allogeneic stem cell transplantation. Furthermore, we revealed a scenario of HIV-1 dynamic different from the commonly described ones. Analysis of viral evolution shows the decrease of viral diversity even during episodes with bursts in viral load.

## Background

Allogeneic stem cell transplantation is frequently used to treat hematologic neoplasms and is a feasible treatment option for patients also infected with human immunodeficiency virus (HIV). Infection of the target cell with HIV-1 requires the presence of the CD4 receptor and a chemokine receptor, mostly either CCR5 (R5) or CXCR4 (X4). Primary HIV-1 infection usually arises from R5-tropic HIV strains. In around 50% of the patients infected with a HIV-1 subtype B virus, a switch in viral co-receptor usage from R5 to X4 can be observed and the presence of X4-tropic HIV is usually accompanied by a faster disease progression [1]. The cause of the coreceptor switch is still unknown, but it can be regarded as a side-effect of selective sweeps in response to evolutionary pressures imposed by the immune response and/or by anti-retroviral therapy (ART). Within-patient HIV evolution is a complex process that is influenced by a multitude of host-, virus- or therapy-specific factors and usually proceeds towards increased diversity of viral isolates with time [2]. Therefore we were interested in analyzing the HIV-1 evolutionary dynamics in samples isolated diachronically from a HIV-infected patient with severe aplastic anemia who underwent stem cell transplantation and was further monitored for 384 days. The viral-host interaction in this patient can be thus considered as occurring after a partial restart of the adaptive immune response due to the stem cell transplantation thereby resembling a primary HIV infection.

## Methods

### Stem cell transplantation

The 34 year-old HIV-infected male had been diagnosed with an HIV-infection in 2001 and with severe aplastic anemia in 2005. The patient had regularly followed highly active antiretroviral therapy (HAART) for 50 months before transplantation and was treated with an allogeneic stem cell transplant of a 10/10 alleles HLA-matched. HAART was discontinued on day 0 until day 34 to avoid potential drug interactions. The conditioning regimen included fludarabine and cyclophosphamide [3]. Blood samples were supplied anonymously by TW who obtained written informed consent from the patient for publication of this case report.

### Sequence analysis of *Env *V3 region

Either chromosomal DNA from peripheral blood mononuclear cells (PBMCs) or viral RNA was used as template. RNA was prepared from the cell-free supernatant of infected cell cultures using QIAamp viral RNA kit (Quiagen, Hilden, Germany) and reverse transcribed using the First strand cDNA Synthesis Kit (Amersham Biosciences, UK). PCR amplification of the V3 region was performed as previously described [4]. Amplification products were directly ligated into the pGEMT vector (Promega, Mannheim, Germany) and nucleotide sequencing was performed using dye-labeled terminators (Eurofins/MWG/operon; Ebersberg, Germany). For each sample ten separate sequences were determined and can be obtained on request.

### Phenotypic determination of coreceptor usage

Virus-containing supernatants were serially diluted in media and aliquoted (50 μL per well, three replicates per dilution) into U-well microtiter plates containing 2 × 10^5 ^of IsnoR5 cells or 4.5 × 10^5 ^PBMC from healthy blood donors in 150 μL medium. After 14 days of incubation at 37°C, cultures were centrifuged and cell-free supernatants were inactivated by the addition of Tween 20 to a final concentration of 0.2%. 25 μL of each sample was transferred to the corresponding well of an ELISA plate for detection of viral Gag protein using a p24 antigen capture assay [4].

TZM-bl cells were obtained from the NIH AIDS Research and Reference Reagent Program (No. 8129). This is a genetically engineered HeLa cell clone that expresses CD4, CXCR4, and CCR5 and contains Tat-responsive reporter genes for firefly luciferase and *Escherichia coli *ß-galactosidase under regulatory control of an HIV-1 long terminal repeat. 1 × 10^6 ^TZM-bl cells were seeded in 6 well- plates (Sarstedt) in presence or absence of 1.5 μM Vicrivroc (Selleck). One day after plating media was renewed and cells were infected with the different HIV isolates. Infection of TZM-bl cells was assessed 2 days after infection by fixing in cold 2% paraformaldehyde in PBS. Afterwards cells were stained for one hour at 37°C by adding 5-bromo-4-chloro-3-indolyl-ß-galactopyranoside (X-Gal) substrate (Roth) at 0.5 mg/ml in PBS containing 4 mM potassium ferrocyanide, 4 mM potassium ferricyanide, 2 mM magnesium chloride. Blue-stained cells/syncytia were counted.

### Humoral immune responses

The presence of antibodies directed against HIV-1 or HIV-2 antigens in plasma samples of the patient were detected with MP Diagnostics HIV BLOT Version 2.2 Western Blots (Illkirch, France)) following the description of the manufacturer.

### *In silico *coreceptor prediction

The coreceptor prediction was done either with the WebPSSM http://indra.mullins.microbiol.washington.edu/webpssm/[5] or the geno2pheno http://coreceptor.bioinf.mpi-inf.mpg.de/index.php[6] software tools [7]. WebPSSM is a bioinformatic tool for predicting HIV-1 coreceptor use from the amino acid sequence of the V3 loop of the envelope gene in subtype B and subtype C HIV. Sequences are aligned against a consensus sequence and their amino acid composition is evaluated on the basis of a position specific scoring matrix (PSSM) which contains information about the amino acid composition of the V3 loop in R5 and X4 viruses. A positive score indicates X4 tropism while negative values can predict X4 virus with high uncertainty only.

Geno2pheno is based on a statistical learning method called a support vector machine (SVM). The program is only trained to detect CXCR4 use, in which X4 and R5X4 viruses are taken as belonging to the X4 class. All predictions were done applying the default FPR of 10%.

### Phylogenetic analysis

V3 sequences were aligned at the amino acid level with MUSCLE and corrected by hand. Bayesian phylogenetic analysis was performed at the nucleotide level with BEAST v1.4.8 http://beast.bio.ed.ac.uk[8] using the GTR+Γ4 model of evolution for both strict clock and uncorrelated log normal relaxed clock, introducing three partitions that corresponded to each of the codon positions, and unlinking parameters across codon positions. Exploratory runs were performed with 50 million steps, writing every 1,000 steps, both for the relaxed and strict clock and compared by calculating the Bayes factor on the corresponding likelihoods. There was better support for the relaxed clock calculations (log Bayes factor = 24.2 ± 0.44). A chain of 100 million steps was therefore calculated for a relaxed clock model, writing every 1,000 steps (later reduced to every 2,000 steps for further analysis), and analyzed with a burn-in of ten million steps. Effective viral population sizes were calculated using the time-labeled sequences gained from the patient, as a "Bayesian skyline" [9]. Maximum Likelihood (ML) phylogenetic analysis was performed with RAxML HPC [10,11] using the GTR+Γ4 model of evolution and the CAT approximation of rate heterogeneity [10], introducing three partitions that corresponded to each of the codon positions. Pair-wise distances between sequences collected at the same day were calculated with the same ML parameters using the best ML tree.

## Case presentation

**Figure 1:**
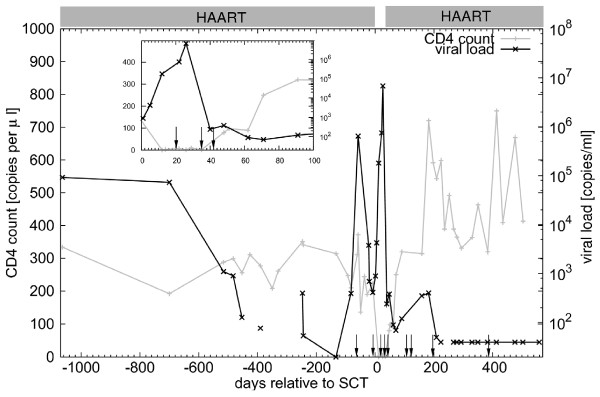
**Viral load and CD4 counts in the stem cell transplanted patient**. The gray bars indicate the duration of ART, which was discontinued at day 0 for allogenic SCT and was restarted at day 34, the inset shows the first 100 days after allo-SCT. Additional sequence samples for phylogenetic analysis were taken at time points marked by arrows. CD4 counts are given in gray and viral load in black.

The 34 year-old HIV-infected male was diagnosed with severe aplastic anemia in 2005. The patient was treated with an allogeneic stem cell transplant and HAART was discontinued on day 0 until day 34. As indicated in figure 1, HIV viral load rapidly rose from undetectable levels to 6.8 × 10^6 ^copies/mL after transplantation and antiretroviral therapy was reintroduced at day 34, resulting in a prompt 4-log reduction of the viral load, and gradually normalizing CD4 counts. A 99% donor chimerism was determined from bone marrow samples and the patient is in CDC-stage A2 now. A progression of his HIV infection could not be noted throughout the procedure [3].

### Genotyping of HIV-1 coreceptor use

**Figure 2:**
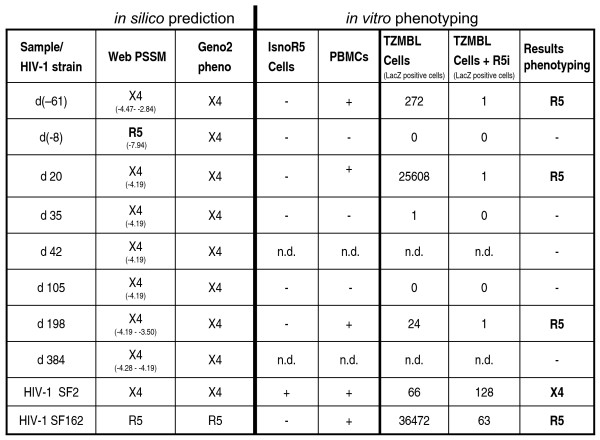
**Summary of genotypic coreceptor predictions and phenotypic coreceptor determinations**. The HIV-1 env C2V5 sequence was amplified from genomic DNA isolated from patient derived PBMCs at the indicated time points as described before [4]. Coreceptor usage was predicted either with WebPSSM (score of sequences shown) or geno2pheno. Patient derived HIV-1 was amplified once on donor PBMCs and was used to infect PBMCs or IsnoR5 cells. Viral replication in the analyzed cells was determined by p24 Elisa [4] and is indicated as + or -. In a second confirmatory assay, patient-derived virus or lab strains were used to infect TZM-bl cells or TZM-bl cells in the presence of Vicrivroc (R5i) and infection was monitored by lacZ staining. Positive cells were counted and the numbers are indicated. The last column gives the coreceptor use deduced from *in vitro *phenotyping. N.d. = not done.

The genotypic coreceptor prediction was done by sequence analysis of the third variable loop (V3) region of the viral envelope (env) gene, which is the primary determinant of coreceptor use. Env sequences were derived of cellular genomic DNA (except for sample at day 198 after transplantation, where cDNA from viral supernatants was isolated) isolated from the patient at different time points as indicated in figure 2 with the date of SCT as reference day 0. Two different algorithms were used for *in silico *coreceptor prediction: WebPSSM [5] and geno2pheno [7]. All *env *sequences were classified as subtype B HIV-1 by the geno2pheno tool.

WebPSSM predicted CXCR4 use except for one sample obtained from the patient. However the negative score of the prediction implied a high degree of uncertainty (figure 2). Geno2pheno predicted for all samples X4 use (figure 2, left).

In addition to the V3 region, sequence changes within the V1, V2 and C4 regions of the envelope protein have also been shown to have a profound impact on coreceptor use (reviewed in [12]) and coreceptor predictions by bioinformatic tools still encounter limitations as R5 and X4 viruses are not trivially separable on the basis of their V3 loop amino acid sequence [13,14]. Therefore we characterized the patient-derived HIV-1 isolates for coreceptor tropism with two cell-based assays. Patient-derived viruses were amplified by one passage in PBMCs of an unrelated donor and for three samples sufficient amounts of virus could be obtained for testing. The coreceptor use was determined by comparison of viral growth in the recently established CCR5-deficient CD4-positive T cell line, IsnoR5 and PBMCs in triplicates [4]. Viral replication in IsnoR5 cells indicates X4 or alternative receptor use, whereas absence of replication in IsnoR5 cells reveals R5 use. The viruses were able to replicate in PBMCs but not in IsnoR5 cells indicating R5 use (figure 2, columns 4 and 5). To verify these results, we used TZM-bl cells, which are a genetically engineered HeLa cell clone that expresses CD4, CXCR4, and CCR5 and contains a Tat-responsive ß-galactosidase gene and allows for monitoring of HIV-1 infection by staining the cells for ß-galactosidase activity. In addition, we analyzed infection of TZM-bl cells in the presence of Vicrivroc, a specific CCR5 inhibitor. TZM-bl cells could be infected with the patient-derived virus, but infection was inhibited in the presence of Vicrivroc (R5i), demonstrating R5 use. For control, we used the typical R5-tropic HIV-1 SF162 and infection of TZM-bl cells was also diminished by Vicrivroc, whereas a typical X4 virus (SF2) was not significantly affected (figure 2 columns 6 and 7).

The data clearly show that in contrast to the *in silico *predictions virions isolated from the patient both before and after the SCT use the CCR5 receptor for entry.

### Humoral immune response

**Figure 3:**
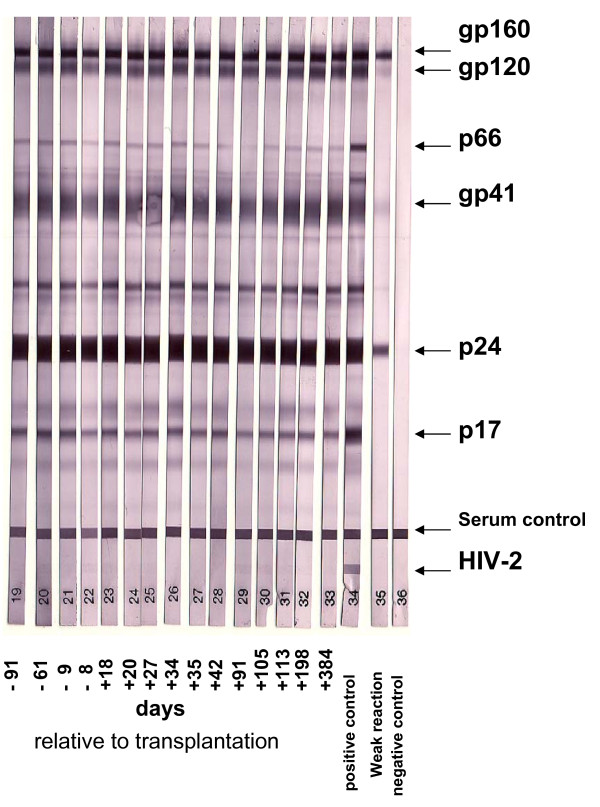
**Humoral immunity**. HIV-1-specific antibodies were detected by Western blot analysis (MP Diagnostics HIV BLOT Version 2.2 Western Blots (Illkirch, France)) from plasma samples at the indicated time points. The major HIV-1 proteins are indicated by arrows and negative, positive and week positive control samples were included.

The immunoblot analysis of HIV antigens revealed the presence of HIV-1 specific antibodies throughout days -92 to +384 after stem cell transplantation. The patient had antibodies reactive towards HIV-1 proteins and was HIV-2 negative (figure 3).

### Intrapatient HIV evolution

The here described SCT can be considered as a treatment interruption in combination with an extensive depletion of the cellular immune response which resulted in a strong viral rebound. Still, HIV specific antibodies remain prevalent in the patient and CD4 cell counts stabilize with the control of viral load after re-initiation of HAART. To analyze viral dynamics in this specific setting, we have studied viral diversity and phylogeny by means of ML and Bayesian methods. We have reconstructed the phylogeny of HIV in the patient assuming viral evolution following a general time-reversible model allowing for heterogeneity in substitution rates among codon positions and sites and incorporating an uncorrelated relaxed clock for the branch rates. In a Bayesian MCMC framework implemented in the software Beast, these parameters and associated tree topologies are sampled and optimized to maximize posterior probabilities given the evidence of sequence samples taken from the patient (d-61, d-8, d20, d35, d42, d105, d120, d198, d384 after transplantation). Time-labeled sequences are used to estimate diachronic changes in effective viral population sizes from its posterior distribution. This so called skyline plot is a proxy for the diversity of the viral population over time [9].

**Figure 4:**
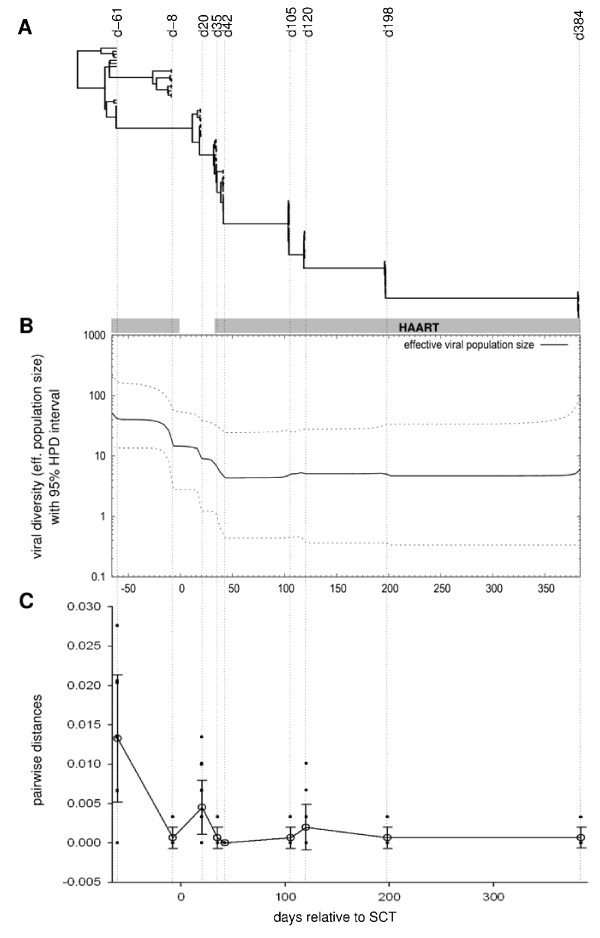
**Viral evolution**. A) Bayesian evolutionary chronogram for the time-labelled viral sequences sampled at the indicated dates. Note that the evolutionary tree evolves towards simplification and finishes simply as a backbone of a single branch. B) Viral effective population sizes as inferred after Bayesian skyline calculation; median value is shown in continuous line; dotted lines represent the 95% highest posterior density (HPD) interval around the median. Viral diversity decreases with time and plateaus after SCT. C) Pair-wise distances among sequences sampled at the corresponding dates, as inferred after maximum likelihood criterion using the GTR+Γ4 model.

Additionally, pair-wise distances based on ML analyses were calculated for the sequences gathered at each time point. A summary of this analysis can be found in figure 4.

Interruption of HAART before SCT treatment results in a burst in viral load (figure 1, inset). However, we could not observe an increase in viral diversity or the emergence of a multitude of strains from reservoirs as a consequence of strong viral replication at the release of viral control. To the contrary, phylogenetic analysis showed that viral diversity decreased after SCT and remained at low levels thereafter. This can be seen qualitatively in the shape of the tree (figure 4A) that is reduced to a backbone of one branch after SCT and more quantitatively in the associated skyline plot (figure 4B) in which the effective population size shrinks to a few sequences indicating minimal diversity and in the decreasing values for pair-wise distances among contemporaneous sequences (figure 4C).

## Discussion

One of the unsolved mysteries of HIV-1 infection is the observation that most HIV-1 transmissions result in a R5 virus infection. Only with time, X4 variants arise in approximately 50% of infected individuals and their appearance correlates with unfavourable clinical outcome [1]. We described here the analysis of the viral rebound of a HIV-infected patient after discontinuation of HAART as a result of bone marrow transplantation. Although genotypic analyses predicted the presence X4-tropic viruses in the patient, phenotypic coreceptor determination revealed only the presence of R5-tropic viruses before and after SCT including one sample taken during the viral rebound. The discrepancy between geno- and phenotyping is most likely due to the fact that the positions 9-10 of the V3 loop, unusually encode for the amino acids arginine and lysine (RK) or lysine and lysine (KK), however the sequences of the patient had an asparagine and lysine (NK) at this position, which could hamper the predictions [7]. Phenotypic coreceptor determination using IsnoR5 cells is highly sensitive and is able to detect minor amounts of X4-tropic HIV, suggesting that no X4 viruses were present in the patient [4]. This is reminiscent to the recently described stem cell transplantation with donor cells homozygous for CCR5delta32 [15]. In this case, putative minor populations of X4-tropic HIV did not cause a re-infection of the patient even two years after transplantation. This indicates that new infection of the donor lymphocytes is either limited to R5-tropic strains or replication of the less abundant X4-tropic strains has not yet been detected [15]. In addition, structured treatment interruption (STI) of chronically HIV-1 infected patients has been described to lead to a rapid re-emergence of HIV from reservoirs and to a dominance of R5- over X4-using strains in patients [16]. This suggests that R5-tropic strains have a selection advantage after stem cell transplantation and STI and that the preferred infection via R5 is independent of the way of entry into the body. This implies that CCR5 inhibitors might be beneficial during the transplantation procedure. In addition, stem cell gene modification, inactivating CCR5 receptor expression, might be considered to be useful, too.

The patient described here had successfully undergone stem cell transplantation and showed a 99% chimerism, which should have eliminated most preexisting HIV-specific effector T cells. Despite the strong decrease in CD4 counts, HIV-specific antibodies were present during stem cell transplantation, which might be caused by long-lived plasma cells that survived the conditioning and the immunosuppressive treatments. The data suggest that the high prevalence of HIV-specific antibodies during viral rebound was unable to prevent rapid HIV replication, but we cannot exclude that they contributed to restrict viral evolution.

The viral envelope protein is exposed to the surface and is subject to selection pressure by the immune system and the rate of evolution of this gene is approximately 1% per year [17]. Both the immune system and antiretroviral therapy impose a selection pressure on the virus towards the emergence of resistant viral strains that usually decrease viral replicative fitness. Thus, viral strains can re-emerge from hidden viral reservoirs in patients as soon as therapy- or immune response-mediated selective pressures are released [18]. This has especially been investigated for STI which mostly leads to a viral rebound associated to the re-emergence of strains and the development of resistances and often linked to a decline in CD4 cell counts [16,19,20]. Surprisingly, the typical pattern of increased viral load and increased viral diversity found in STI does not occur in our case. While HAART interruption does result in a viral rebound, there is no increased diversity of viral strains that might be released from the patient's hidden reservoirs. This indicates that the, at least partial knock-out of the immune system and the establishment of a new immune system imposes a selection pressure different from those generated by STI.

One might hypothesize that viral load is controlled by therapeutic measures before the patient's immune system is again operational after SCT. Therefore no specific immune response could be newly established shifting the relevance of evolutionary pressures towards the less strain specific therapeutic measures. This might decrease the selection pressure for diversity in the viral quasispecies [21,22]. However, this does not resolve the puzzle why HIV shows less diversity in this patient after SCT and STI than under STI alone. It would be anticipated instead that the additional depletion of the cellular immune system should worsen the ambivalent prognosis after STI. Maybe elimination of hidden HIV-1 reservoirs before SCT suffices to reduce the potential to generate viral diversity from dormant sequences, implying that viral diversity arising de novo through viral evolution is limited.

## Conclusion

In summary, our data from this case report of an HIV-1-infected patient undergoing SCT evidence that only R5-tropic virus was found in the patient before and after transplantation. In addition, the presence of HIV-1-specific antibodies was found in all plasma samples over time. Remarkably, the patient experienced an HIV-1 dynamic scenario different from commonly described ones. Detailed analysis of viral evolution shows the decrease of viral diversity even during episodes with bursts of viral load in contrast to observations under STI.

Given that SCT has become a more frequent treatment option for HIV infected patients, it will be important to investigate in future studies whether our observations of only one patient are rather anecdotal evidence or point instead towards an important mechanism of HIV evolution. In addition, our findings might have implications for vaccine development, which is mainly hampered by the great diversity of HIV.

## Consent

Written informed consent was obtained by TW from the patient for publication of this case report and any accompanying images. A copy of the written consent is available for review by the Editor-in-Chief of this journal upon request.

## Competing interests

The authors declare that they have no competing interests.

## Authors' contributions

BeK, BiK, BN, GW carried out the molecular genetic studies and participated in the genotypic coreceptor predictions; AT carried out the genotypic coreceptor predictions and helped to draft the manuscript; CK and IGB performed the bioinformatic analysis and helped to draft the manuscript. TW and AW conceived the study, and participated in its design and coordination. BSS conceived and coordinated the study and drafted the manuscript. All authors read and approved the final manuscript.
